# Risk of Early Childhood Caries Estimated by Maternal Dental Caries during Pregnancy: A Retrospective Cohort Study

**DOI:** 10.1055/s-0043-1769896

**Published:** 2024-03-22

**Authors:** Sunithi Thearawiboon, Chanapong Rojanaworarit

**Affiliations:** 1 Dental Department, Prachathipat Hospital, Pathum Thani, Thailand; 2 Department of Population Health, School of Health Professions and Human Services, Hofstra University, Hempstead, New York, United States

**Keywords:** dental caries, risk indicator, pediatric dentistry, epidemiology

## Abstract

**Objective** Public policy promoting prenatal dental care to provide long-term prevention of early childhood caries (ECC) in offspring would require evidence regarding the ECC risk associated with maternal dental caries during pregnancy. This study evaluated that association using a design capable of assessing temporal relationships and considered a directed acyclic graph to guide the adjustment of pertinent confounders.

**Materials and Methods** This retrospective cohort study analyzed data from 158 mother–child dyads attending care at Prachathipat Hospital, Pathum Thani, Thailand. Maternal dental caries data at their first visits to prenatal oral care from February 2012 to May 2017 were traced forwardly to match the oral health data of offspring who visited the hospital from May 2013 to March 2018.

**Results** Univariable and multivariable fractional logit regression models along with the calculation of average marginal effects revealed that children born to two categories of mothers with 1 to 5 and ≥6 carious teeth during pregnancy would averagely develop 4.5 to 5 and 7.9 to 8.8 more carious teeth per 100 teeth than dental caries would occur in offspring of caries-free mothers.

**Conclusion** This evidence identified the role of maternal dental caries during pregnancy as a significant clinical risk indicator for ECC and supported the provision of prenatal dental care for mothers to prevent ECC in offspring.

## Introduction

Early childhood caries (ECC) is a disease caused by an interaction of cariogenic bacteria and other factors that occur in deciduous teeth of children aged under 6 years of age. 1 2 Severe negative effects of ECC include odontogenic pain, masticatory dysfunction, malnourishment, undergrowth, and decreased quality of life. 3 4 5 Various disciplines have contributed to the understanding of ECC etiology. Maternal transmission could be a route by which infants are inoculated with mutans streptococci. 6 7 Nonetheless, mutans streptococci genotypes detected in children did not necessarily match their maternal strains. 8 Another elucidation for the ECC etiology emphasizes the interrelationship of multiple risk factors. 8 9 For example, inadequate prenatal oral health education may contribute to mothers' lack of competency to care for their offspring's oral health which can impact the individual risk of caries among children. 10 From an epidemiological perspective, the application of directed acyclic graph (DAG) 11 12 has deepened the understanding of various roles of factors—such as confounder and collider—that interplay in the ECC etiology. 13 14 This novel approach improves the validity of the effect estimate of an explanatory variable on the outcome by enabling rational specification of variables to be controlled for confounding and preventing biases. 12 Nonetheless, the application of DAG to measure the effects of factors affecting ECC is still scarce. 13 14

The link between maternal dental caries and ECC in their offspring has been supported by multidisciplinary research. 14 15 16 17 Maternal dental caries can be a source of cariogenic bacteria that mothers pass onto their offspring. 6 7 Maternal caries can also reflect the maternal socioeconomic status and oral health behavior which may impact oral hygiene practices and the ECC risk of a child. 18 Epidemiological studies have also demonstrated that maternal caries experience was related to the offspring's ECC. 19 20 Nevertheless, the evidence was mainly obtained from cross-sectional studies. 15 16 19 20 Investigations of the occurrence of ECC concerning maternal caries during pregnancy, which is a critical period for prenatal oral care and prenatal ECC prevention, 21 are in need.

This study evaluated the association between maternal dental caries during pregnancy and the ECC risk in offspring among Thai mother–child dyads attending a public dental service provided by a district hospital. The findings would not only serve as epidemiological evidence regarding the role of maternal caries during pregnancy as a clinical risk indicator for ECC in children but also inform public policy regarding the need to strengthen prenatal oral health care to achieve the long-term goal of ECC prevention in children.

## Materials and Methods

### Study Design and Study Participants

A retrospective cohort design was applied to collect service-based data from records at Prachathipat Hospital—a community hospital providing primary and secondary medical care in Thanyaburi District, Pathum Thani Province, Thailand. Thai women with singleton pregnancies attending the integrated services of antenatal and oral health care at this health facility from February 2012 to May 2017 and who later gave live births were eligible for the cohort inception. The maternal data were matched to their offspring's general and oral health-related data who later visited this facility for the integrated services of childhood immunization and oral health promotion from May 2013 to March 2018.

A minimal study size of 105 mother–child dyads for this cohort study was estimated assuming an α of 0.05 and statistical power of 80%. Based on the past service context at this facility, the ratio of caries-free mothers to their counterparts was estimated at 3:7 or 0.43. Presumably, 1 in 20 or 5% of children born to caries-free mothers might develop dental caries, while up to 3 in 10 or 30% of those born to mothers with caries might develop caries. Ultimately, data from all 158 mother–child dyads were obtained for analysis.

### Outcome Measurement

The outcome was defined as “risk of ECC” (π) calculated as a probability value that the numerator is the count number of the carious teeth (whether they were restored or not) plus missing teeth due to caries and divided by the denominator of the count number of all ever erupted teeth at risk of caries in the follow-up period, and multiplied by 100 to obtain a percentage. For instance, a 22-month-old child with 2 carious teeth from a total of 16 ever-erupted teeth would have the π value equal (2/16) × 100% or 12.5%. The magnitude of π closer to 100% implies a higher ECC risk in terms of the proportion of carious teeth from the whole number of erupted teeth. This outcome measurement was used instead of the count number of carious teeth due to the nature of the dynamic cohort in terms of both age and tooth eruption.

Oral examination to determine a child's caries status was routinely undertaken by one dentist (S.T.) and two dental hygienists during the child's visits for the integrated services of childhood immunization and oral health promotion at the hospital. Starting from the age of 2 months, each child was scheduled for a series of vaccinations and each visit was followed by an oral health service at the dental department. From the age of 9 months, oral examination to determine caries status was added to every vaccination visit. The method of oral examination used for this service adopted the practical technique of the knee-to-knee oral examination procedure undertaken with assistance from the caregiver. 22 Visual inspection of the oral cavity under natural light with the use of a mouth mirror was used to determine the number of erupted teeth and carious teeth. Intra- and interexaminer calibration for caries detection among the dental service providers has been performed on a yearly basis as a part of the annual intra- and interexaminer calibration and dental service development training program for dental personnel of public hospitals organized by Pathum Thani Provincial Public Health Office. The calibration was undertaken using multiple selected standard cases. The findings were of high agreement, and a few cases with mismatched examination results were reviewed and reexamined.

Data obtained from the oral examination were recorded visit-by-visit in the hospital's dental charts. Since this study leveraged these retrospective dental records of the real-world public dental service that prioritized identification of cavitated caries in need of restoration during hospital visits before routine fluoride varnish application in all children; these records, therefore, represented importantly cavitated caries. To obtain the outcome data for this study, a researcher (S.T.) examined each child's dental records from all available dental visits during the follow-up period.

### Exposure Measurement

Maternal characteristics were routinely determined in the mothers' first visit to this facility's integrated antenatal and oral health care services. Maternal dental caries in this study was defined as the number of carious teeth, whether restorable or requiring extraction. Oral examination to determine maternal caries was determined by one dentist (S.T.). Unlike the conventional Decayed, Missing, and Filled Teeth (DMFT) index that measures past caries experience, this variable instead reflected active caries during pregnancy. Data regarding maternal age, oral health insurance type, and maternal dental caries were retrieved from the hospital electronic database (HosXP).

All variables related to child's characteristics, risk behaviors, and oral hygiene practices were routinely recorded using the standard data record form of the Bureau of Dental Health, Ministry of Public Health, Thailand. The caregiver who brought the child for the oral health promotion service was interviewed during all visits during the study period from February 2012 to May 2017. For each child, records from all visits were reviewed by the researcher (S.T.).

### Statistical Analyses

Descriptive statistics were used to summarize the characteristics of study participants. To assess the crude and adjusted estimates of the association between maternal dental caries during pregnancy and the risk of ECC, fractional response generalized linear regression with logit link function and robust standard errors (or fractional logit regression model) was applied. 23 24 The dependent variable was the risk of childhood caries (π), which was a fractional response variable having values ranging from 0 (caries free) to 1 (all ever-erupted teeth affected by caries). The major independent variable was the number of maternal dental caries during pregnancy, which was categorized into 3 categories comprising 0 (caries-free), 1 to 5, and ≥6 teeth. Categorization of the number of maternal caries was adapted from the previous study which used ≤5 DMFT to represent low caries experience and ≥6 DMFT to represent medium to high caries experience. 14 Since π can have values ranging from 0 to 1, the variance tends to decrease when the mean gets closer to one of the boundaries (e.g., closer to π = 0 if the majority of children were caries free) and the effect of the independent variable tended to be nonlinear. Unlike the ordinary least square regression method which estimates a coefficient or a slope of a linear regression line that represents a single marginal effect of one unit increase in an independent variable ( *X* ) on a probability that an outcome ( *Y* ) is equal to 1 as opposed to 0; a coefficient estimated from the fractional logit model neither implies a single marginal effect nor the interpretation of the effect of a one-unit change in *X* directly on *Y* ^25^ , but rather on an index. The coefficient estimated by the logit model is not straightforwardly interpretable because there is not just one marginal effect of *X* . The effect of *X* can vary depending on every independent variable in the regression function. Given the following regression function of *E* ( *Y* | *X* , *Z* ) = Logistic (β _0_ + β _1_ X + β _2_ Z), where *X* and *Z* are independent variables and *Y* is the outcome. The marginal effect of *X* is estimated by the derivative of the function as ∂ *Y* /∂ *X* = β _1_ Logistic (β _0_ + β _1_ X + β _2_ Z)(1 *−* Logistic [β _0_ + β _1_ X + β _2_ Z]). The term “Logistic (β _0_ + β _1_ X + β _2_ Z)(1 *−* Logistic [β _0_ + β _1_ X + β _2_ Z])” is regarded as the index. From this illustration, the marginal effect of *X* or ∂ *Y* /∂ *X* is not a constant value or equal to the β _1_ coefficient alone, but rather the β _1_ multiplied by the index term. This results in varying multiple marginal effects of *X* depending on the values of all independent variables (e.g., *Z* ) in the regression model. 25 Therefore, the coefficient estimated from the fractional logit model would only provide information regarding the direction of the effect of *X* (either positive or negative) and statistical significance, but not the magnitude of the effect. To provide a single summary of the varying marginal effects of *X* , the average marginal effect (AME)—or the average of all marginal effects calculated from all observations–was calculated from the prediction based on the previously fitted fractional logit model. 26

**Fig. 1:**
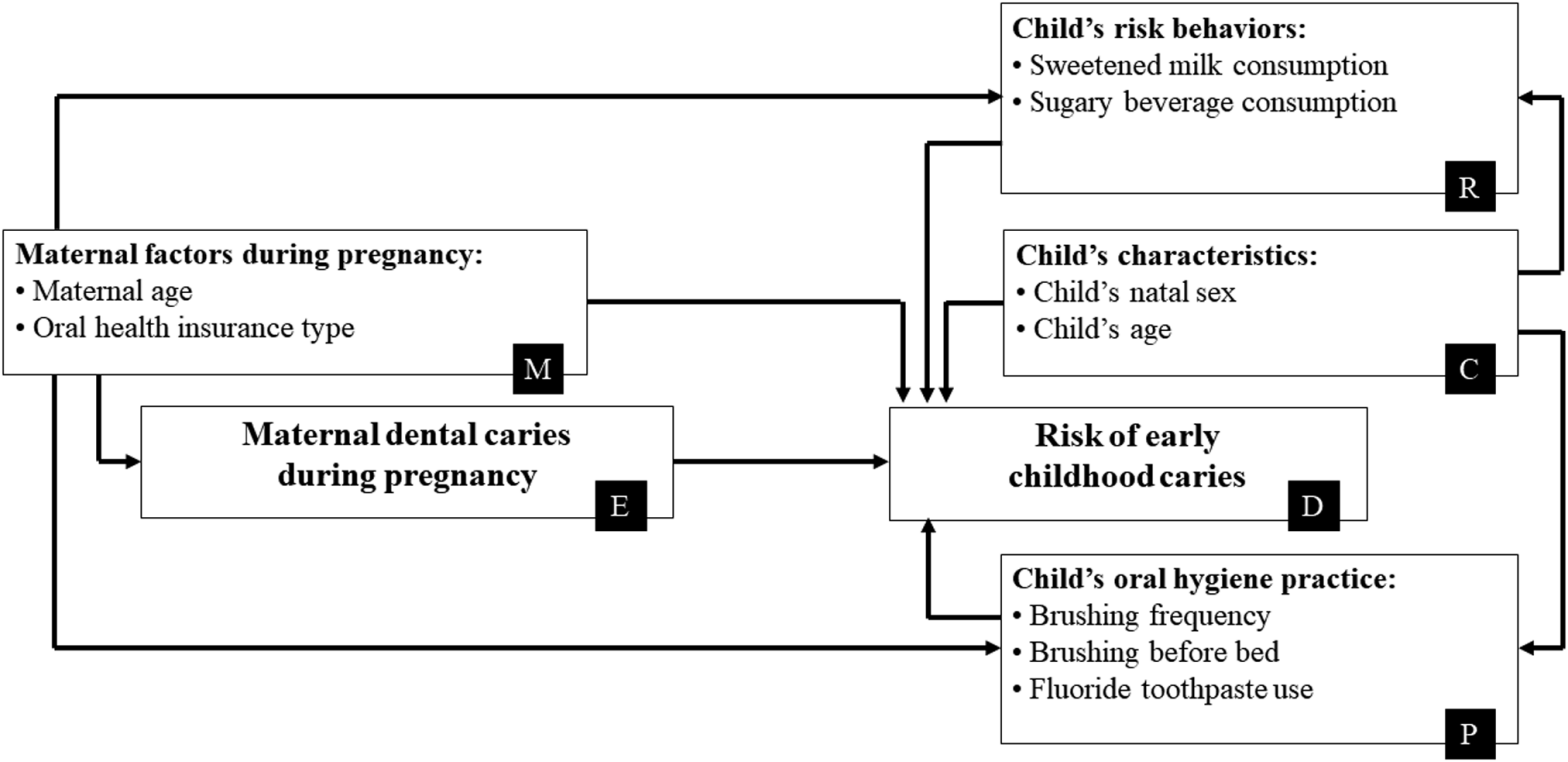
Statistical models according to different paths of directed acyclic graphs. **Model 1.** Unadjusted model of maternal dental caries (E) and risk of early childhood caries (D): E → D; **Model 2.** Model of maternal dental caries (E) and risk of ECC (D) adjusted for maternal factors during pregnancy (M): E ← M → D; **Model 3.** Model of maternal dental caries (E) and risk of ECC (D) adjusted for maternal factors (M), and child's characteristics (C), and child's risk behaviors (R): E ← M → R ← C → D; **Model 4.** Model of maternal dental caries (E) and risk of ECC (D) adjusted for maternal factors (M), and child's characteristics (C), and child's oral hygiene practice (P): E ← M → P ← C → D; **Model 5.** Model of maternal dental caries (E) and risk of ECC (D) adjusted for maternal factors (M), and child's characteristics (C), and child's risk behaviors (R), and child's oral hygiene practice (P): E ← M → R & P ← C → D.

The multivariable regression analyses were sequentially executed according to an epidemiological causal model depicted by a DAG ( Fig. 1 ). The dependent variable (D) was π. Model 1 was an unadjusted (crude) model that included only one independent variable of maternal dental caries during pregnancy—the main exposure (E). Model 2 included (E) and additionally adjusted for a set of maternal factors during pregnancy (M) which included maternal age and maternal oral health insurance type. Independent variables in Model 3 comprised (E), (M), and additionally adjusted for a set of child's characteristics (C) which were the child's natal sex and age, and a set of child's risk behaviors (R) including sweetened milk consumption and sugary beverage consumption. Model 4 comprised (E), (M), (C) and additionally adjusted for a set of child's oral hygiene practice factors (P) including brushing frequency, brushing before bed, and fluoride toothpaste use. Model 5 analyzed the main exposure (E) adjusting for all other covariates: (M), (C), (R), and (P).

## Results

At the first visit to antenatal care, the average age of all 158 mothers was 22.7 years old, and around one-third of them were pregnant under the age of 20 years. Although most mothers had their prenatal oral health care financially supported by public health insurance programs, there were still 14 women who did not. The average number of carious teeth among all the mothers was 4.4 teeth and slightly over half of them (53.8%) had 1 to 5 carious teeth. At the end of the follow-up period in March 2018, the children's age ranged from 9 to 47 months, with a median age of 20.5 months. Most of the children (79.1%) remained caries-free throughout the follow-up period, although 15.2 and 5.7% had 1 to 25% and >25% risk of ECC, respectively. One child was identified as having a 100% risk of ECC, which meant that caries damaged all his ever-erupted teeth. In comparison to the children with 1 to 25% and >25% risk of ECC, those who remained caries free were born to mothers with significantly fewer maternal caries during pregnancy ( *p* < 0.001). Additionally, all 21 women who were caries free had children who remained caries free throughout the follow-up period. Children with sweetened milk consumption had significantly greater proportions of them at higher levels of ECC risk than those without the consumption ( *p* = 0.021). The significantly greater proportions of children having 1 to 25% and >25% ECC risk were also found among those with sugary beverage consumption compared with their counterparts ( *p* = 0.013; Table 1 ).

**Table 1:** Characteristics of mothers and children stratified by risk of early childhood caries (%)

Characteristics	Total *n* (%) a		Risk of early childhood caries (%)						*p-* Value
			Caries free		1–25%		> 25%		
			*n* (%) b		*n* (%) b		*n* (%) b		
Overall	158		125 (79.1)		24 (15.2)		9 (5.7)		
Risk of early childhood caries (%): Mean (SD), 5.3 (13.7); Median (IQR), 0 (0); Min.–Max., 0–100									
Maternal characteristics									
Maternal age (year)									
Mean (SD)	22.7 (5.9)		22.2 (5.3)		24.0 (7.3)		26.0 (8.4)		
Median (IQR)	21 (8)		21 (7)		21 (10)		22 (14)		0.381 c
Min.–Max.	14–41		14–40		16–41		17–41		
< 20	51 (32.3)		42 (82.3)		8 (15.7)		1 (2.0)		
20–35	100 (63.3)		80 (80.0)		13 (13.0)		7 (7.0)		
> 35	7 (4.4)		3 (42.9)		3 (42.9)		1 (14.2)		
Oral health insurance									
Universal Coverage	126 (79.8)		101 (80.1)		18 (14.3)		7 (5.6)		0.255 d
Social Security Scheme	18 (11.4)		11 (61.1)		5 (27.8)		2 (11.1)		
Out-of-pocket payment	14 (8.8)		13 (92.9)		1 (7.1)		0 (0)		
Maternal dental caries (tooth)									
Mean (SD)	4.4 (3.7)		3.9 (3.4)		6.4 (3.9)		6.8 (4.3)		
Median (IQR)	4 (5)		3 (5) e		7 (4)		5 (5)		<0.001 c
Min.–Max.	0–20		0–19		1–20		2–16		
0	21 (13.3)		21 (100.0)		0 (0)		0 (0)		
1–5	85 (53.8)		70 (82.3)		10 (11.8)		5 (5.9)		
≥6	52 (32.9)		34 (65.4)		14 (26.9)		4 (7.7)		
Child's characteristics									
Sex									
Female	82 (51.9)		66 (80.5)		12 (14.6)		4 (4.9)		0.869 d
Male	76 (48.1)		59 (77.6)		12 (15.8)		5 (6.6)		
Child's age (month)									
Mean (SD)	23.2 (10.2)		21.7 (10.5)		29.3 (6.3)		28.7 (7.3)		
Median (IQR)	20.5 (15)		18 (18) e		31 (8)		30 (8)		<0.001 c
Min.–Max.	9–47		9–47		18–39		18–39		
9–12	34 (21.5)		34 (100.0)		0 (0)		0 (0)		
13–24	49 (31.0)		41 (83.7)		5 (10.2)		3 (6.1)		
25–36	63 (39.9)		40 (63.5)		18 (28.6)		5 (7.9)		
37–47	12 (7.6)		10 (83.4)		1 (8.3)		1 (8.3)		
Child's risk behaviors									
Sweetened milk consumption									
No	126 (79.7)		104 (82.5)		18 (14.3)		4 (3.2)		0.021 d
Yes	32 (20.3)		21 (65.6)		6 (18.8)		5 (15.6)		
Sugary beverage consumption									
No	74 (46.8)		66 (89.2)		6 (8.1)		2 (2.7)		0.013 d
Yes	84 (53.2)		59 (70.2)		18 (21.4)		7 (8.4)		
Bottle feeding									
No	1 (0.6)		1 (100.0)		0 (0)		0 (0)		1.000 d
Yes	157 (99.4)		124 (79.0)		24 (15.3)		9 (5.7)		
Child's oral hygiene practice									
Brushing frequency (time/day)									
Mean (SD)	1.3 (0.8)		1.3 (0.7)		1.5 (0.8)		1 (0.9)		
Median (IQR)	1 (1)		1 (1)		2 (1)		1 (1)		0.240 c
Min.–Max.	0–3		0–3		0–3		0–2		
≤1	86 (54.4)		70 (81.4)		10 (11.6)		6 (7.0)		
> 1	72 (45.6)		55 (76.4)		14 (19.4)		3 (4.2)		
Brushing before bed									
Yes	83 (52.5)		67 (80.7)		14 (16.9)		2 (2.4)		0.167 d
No	75 (47.5)		58 (77.3)		10 (13.3)		7 (9.4)		
Fluoride toothpaste use									
Yes	98 (62.0)		72 (73.5)		20 (20.4)		6 (6.1)		0.055 d
No	60 (38.0)		53 (88.3)		4 (6.7)		3 (5.0)		

Abbreviations: IQR, interquartile range; Max., maximum; Min., minimum; SD, standard deviation.

a Percentage by column.

b Percentage by row.

c Kruskal-Wallis equality-of-populations rank test.

d Exact probability test.

e Statistically significant difference from the other two subgroups.

Findings from the univariable analyses are presented in Table 2 . In comparison to children born to caries-free mothers, the offspring of mothers having carious teeth were at higher risk of ECC as revealed by positive values of coefficients in both categories of mothers having 1 to 5 and ≥6 carious teeth with statistical significance. According to the AME obtained following the fractional logit regression, the ECC risk among children born to mothers with 1 to 5 carious teeth during pregnancy would rise by 0.045 or 4.5% on average compared with those born to caries-free mothers ( *p* < 0.001). In other words, caries would averagely arise in 4.5 more teeth per 100 ever-erupted teeth of children born to mothers with 1 to 5 carious teeth during pregnancy than it would occur per 100 ever-erupted teeth of children born to mothers without caries. Moreover, compared with children born to women with no caries during pregnancy, the ECC risk for those whose mothers had ≥6 carious teeth would increase by 0.088 or 8.8% on average ( *p* < 0.001). This indicated that caries would averagely arise in 8.8 more teeth per 100 ever-erupted teeth of children born to mothers with ≥6 carious teeth during pregnancy than it would occur per 100 ever-erupted teeth of children born to caries-free mothers.

**Table 2:** Univariable fractional logit regression analysis of association between risk of early childhood caries (π) and each explanatory variable

Risk indicators	Risk of early childhood caries (π)			Univariable analysis a			Marginal effect b		
	Mean (SD)	Median (IQR)	Min.– Max.	Coefficient	95% CI	*p-* Value	dy/dx	95% CI	*p-* Value
Maternal dental caries (tooth)									
0	0 (0)	0 (0)	0–0	Reference	Reference	Reference	Reference	Reference	Reference
1–5	0.04 (0.12)	0 (0)	0–0.57	16.04	14.34, 17.74	<0.001	0.045	0.025, 0.065	<0.001
≥6	0.09 (0.18)	0 (0)	0–1.00	16.77	15.06, 18.47	<0.001	0.088	0.050, 0.127	<0.001
Maternal factors during pregnancy									
Maternal age (year)									
20–35	0.06 (0.16)	0 (0)	0–1.00	Reference	Reference	Reference	Reference	Reference	Reference
< 20	0.03 (0.08)	0 (0)	0–0.31	−0.65	−1.51, 0.21	0.141	−0.027	−0.064, 0.009	0.145
> 35	0.12 (0.18)	0 (0.20)	0–0.50	0.80	−0.48, 2.08	0.218	0.064	−0.065, 0.193	0.329
Oral health insurance type									
Universal Coverage	0.05 (0.14)	0 (0)	0–1.00	Reference	Reference	Reference	Reference	Reference	Reference
Social Security Scheme	0.10 (0.15)	0 (0.17)	0–0.50	0.66	−0.28, 1.59	0.167	0.044	−0.029, 0.116	0.237
Out-of-pocket payment	0.01 (0.04)	0 (0)	0–0.17	−1.51	−3.49, 0.47	0.135	−0.040	−0.073, −0.007	0.019
Child's characteristics									
Sex									
Female	0.05 (0.12)	0 (0)	0–0.60	Reference	Reference	Reference	Reference	Reference	Reference
Male	0.06 (0.15)	0 (0)	0–1.00	0.14	−0.71, 0.98	0.751	0.007	−0.036, 0.050	0.754
Age (month)									
9–12	0 (0)	0 (0)	0–0	Reference	Reference	Reference	Reference	Reference	Reference
13–24	0.04 (0.11)	0 (0)	0–0.57	15.12	14.28, 15.97	<0.001	0.041	0.010, 0.072	0.010
25–36	0.09 (0.18)	0 (0.15)	0–1	16.00	15.41, 16.58	<0.001	0.092	0.049, 0.135	<0.001
37–47	0.05 (0.14)	0 (0)	0–0.5	15.34	13.66, 17.01	<0.001	0.050	−0.028, 0.128	0.212
Child's risk behaviors									
Sweetened milk consumption									
No	0.04 (0.10)	0 (0)	0–0.6	Reference	Reference	Reference	Reference	Reference	Reference
Yes	0.12 (0.23)	0 (0.18)	0–1	1.28	0.40, 2.16	0.004	0.063	0.011, 0.115	0.018
Sugary beverage consumption									
No	0.02 (0.08)	0 (0)	0–0.5	Reference	Reference	Reference	Reference	Reference	Reference
Yes	0.08 (0.17)	0 (0.1)	0–1	1.29	0.35, 2.24	0.007	0.057	0.016, 0.097	0.006
Child's oral hygiene practice									
Brushing frequency (time/day)									
≤1	0.06 (0.15)	0 (0)	0–1	Reference	Reference	Reference	Reference	Reference	Reference
> 1	0.05 (0.12)	0 (0)	0–0.6	−0.10	−0.93, 0.72	0.806	−0.005	−0.047, 0.037	0.807
Brushing before bed									
Yes	0.04 (0.10)	0 (0)	0–0.6	Reference	Reference	Reference	Reference	Reference	Reference
No	0.07 (0.17)	0 (0)	0–1	0.70	−0.18, 1.53	0.097	0.035	−0.009, 0.078	0.118
Fluoride toothpaste use									
Yes	0.07 (0.16)	0 (0.1)	0–1	Reference	Reference	Reference	Reference	Reference	Reference
No	0.03 (0.09)	0 (0)	0–0.4	−0.80	−1.71, 0.11	0.086	−0.036	−0.074, 0.003	0.071

Abbreviation: 95% CI, 95% confidence interval.

a Coefficients estimated by univariable fractional logit regression.

b Postestimation marginal effect (derivative) of covariate on outcome.

Multivariable analyses of the effect of maternal dental caries during pregnancy on the risk of ECC are presented in Table 3 . Model 1 represented the unadjusted effect of maternal caries on offspring's ECC risk, which indicated that children whose mothers had dental caries during pregnancy were significantly at higher risk of ECC compared with those born to caries-free mothers. In addition, as confirmed by all statistically significant positive coefficients in all subsequent multivariable models adjusting for various sets of covariates (Model 2 to 5), children born to mothers with 1 to 5 and ≥6 carious teeth during pregnancy were at increased risk of ECC compared with those of caries-free mothers. Models 1 to 5 consistently indicated that the ECC risk among offspring of mothers with 1 to 5 carious teeth during pregnancy would significantly increase by the range of 0.045 to 0.05 (or 4.5 to 5%) compared with children born to caries-free mothers. In other words, caries would averagely arise in 4.5 to 5 more teeth per 100 ever-erupted teeth of children born to mothers with 1 to 5 carious teeth during pregnancy than it would occur per 100 ever-erupted teeth of children born to mothers without caries. For children whose mothers had ≥6 carious teeth during pregnancy, all five models also consistently identified that their ECC risk would significantly rise by the range of 0.079 to 0.088 (or 7.9% to 8.8%) compared with the children of caries-free mothers. This indicated that caries would averagely arise in 7.9 to 8.8 more teeth per 100 ever-erupted teeth of children born to mothers with ≥6 carious teeth during pregnancy than it would occur per 100 ever-erupted teeth of children born to mothers without caries.

**Table 3:** Multivariable fractional logit regression analyses of association between maternal dental caries and risk of early childhood caries (π)

Risk indicator	Model 1				Model 2				Model 3		
	Coef. (95% CI)	*p* -Value	dy/dx (95% CI)	*p* -Value	Coef. (95% CI)	*p* -Value	dy/dx (95% CI)	*p* -Value	Coef. (95% CI)	*p* -Value	dy/dx (95% CI)	*p* -Value
Maternal dental caries (tooth)												
0	Reference	–	Reference	–	Reference	–	Reference	–	Reference	–	Reference	–
1–5	16.04 (14.34, 17.74)	<0.001	0.045 (0.025, 0.065)	<0.001	15.75 (14.76, 16.74)	<0.001	0.046 (0.017, 0.075)	0.002	15.96 (15.04, 16.89)	<0.001	0.046 (0.008, 0.083)	0.017
≥ 6	16.77 (15.06, 18.47)	<0.001	0.088 (0.050, 0.127)	<0.001	16.41 (15.39, 17.43)	<0.001	0.085 (0.033, 0.137)	0.001	16.58 (15.78, 17.38)	<0.001	0.079 (0.023, 0.134)	0.005
Maternal factors during pregnancy												
Maternal age (year)												
20–35					Reference	–	Reference	–	Reference	–	Reference	–
< 20					−0.69 (−1.57, 0.19)	0.126	−0.029 (−0.068, 0.009)	0.135	−0.71 (−1.56, 0.148)	0.105	−0.029 (−0.066, 0.008)	0.123
> 35					0.27 (−1.41, 1.94)	0.757	0.017 (−0.099, 0.133)	0.777	−0.02 (−1.71, 1.68)	0.985	−0.001 (−0.090, 0.089)	0.985
Oral health insurance type												
Universal Coverage					Reference	–	Reference	–	Reference	–	Reference	–
Social Security Scheme					0.39 (−0.83, 1.62)	0.527	0.023 (−0.056, 0.101)	0.567	0.27 (−1.03, 1.57)	0.684	0.014 (−0.057, 0.085)	0.705
Out-of-pocket payment					−1.38 (−3.24, 0.48)	0.147	−0.039 (−0.075, −0.002)	0.037	−1.02 (−2.95, 0.91)	0.3	−0.032 (−0.077, 0.013)	0.166
Child's characteristics												
Sex												
Female									Reference	–	Reference	–
Male									0.02 (−0.75, 0.80)	0.951	0.001 (−0.035, 0.037)	0.951
Age (month)												
9–12									Reference	–	Reference	–
13–24									15.94 (15.06, 16.81)	<0.001	0.048 (0.010, 0.087)	0.014
25–36									16.46 (15.72, 17.20)	<0.001	0.077 (0.022, 0.131)	0.006
37–47									15.78 (14.34, 17.23)	<0.001	0.042 (−0.020, 0.104)	0.186
Child's risk behaviors												
Sweetened milk consumption												
No									Reference	–	Reference	–
Yes									0.95 (0.09, 1.80)	0.029	0.051 (−0.009, 0.110)	0.096
Sugary beverage consumption												
No									Reference	–	Reference	–
Yes									0.85 (−0.212, 1.91)	0.117	0.035 (−0.007, 0.077)	0.104
Child's oral hygiene practice												
Brushing frequency (time/day)												
≤1												
> 1												
Brushing before bed												
Yes												
No												
Fluoride toothpaste use												
Yes												
No												
Risk indicator	Model 4				Model 5							
	**Coef. (95% CI)**	*p* -Value	dy/dx (95% CI)	*p* -Value	Coef. (95% CI)	*p* -Value	dy/dx (95% CI)	*p* -Value				
Maternal dental caries (tooth)												
0	Reference	–	Reference	–	Reference	–	Reference	–				
1–5	16.66 (15.94, 17.38)	<0.001	0.050 (0.015, 0.086)	0.005	16.10 (15.13, 17.07)	<0.001	0.047 (0.019, 0.074)	0.001				
≥ 6	17.27 (16.26, 18.28)	<0.001	0.085 (0.021, 0.150)	0.009	16.72 (15.73, 17.71)	<0.001	0.080 (0.026, 0.134)	0.004				
Maternal factors during pregnancy												
Maternal age (year)												
20–35	Reference	–	Reference	–	Reference	–	Reference	–				
< 20	−1.03 (−2.01, −0.05)	0.04	−0.042 (−0.087, 0.002)	0.062	−0.79 (−1.64, 0.06)	0.067	−0.033 (−0.070, 0.004)	0.079				
> 35	−0.45 (−2.42, 1.53)	0.657	−0.023 (−0.110, 0.065)	0.611	−0.55 (−2.36, 1.25)	0.548	−0.025 (−0.096, 0.045)	0.485				
Oral health insurance type												
Universal Coverage	Reference	–	Reference	–	Reference	–	Reference	–				
Social Security Scheme	0.64 (−0.57, 1.84)	0.299	0.036 (−0.043, 0.114)	0.371	0.64 (−0.78, 2.06)	0.377	0.034 (−0.054, 0.123)	0.449				
Out-of-pocket payment	−1.01 (−3.10, 1.08)	0.343	−0.030 (−0.077, 0.016)	0.201	−0.73 (−2.88, 1.41)	0.504	−0.024 (−0.078, 0.031)	0.397				
Child's characteristics												
Sex												
Female	Reference	–	Reference	–	Reference	–	Reference	–				
Male	0.20 (−0.64, 1.03)	0.647	0.009 (−0.030, 0.048)	0.648	0.14 (−0.71, 1.00)	0.742	0.006 (−0.032, 0.045)	0.741				
Age (month)												
9–12	Reference	–	Reference	–	Reference	–	Reference	–				
13–24	16.31 (15.40, 17.21)	<0.001	0.049 (0.009, 0.089)	0.016	15.93 (15.07, 16.80)	<0.001	0.046 (0.006, 0.085)	0.024				
25–36	16.86 (16.12, 17.59)	<0.001	0.080 (0.019, 0.141)	0.01	16.52 (15.75, 17.29)	<0.001	0.077 (0.031, 0.122)	0.001				
37–47	16.35 (14.56, 18.13)	<0.001	0.051 (−0.028, 0.130)	0.207	16.06 (14.53, 17.59)	<0.001	0.051 (−0.017, 0.120)	0.141				
Child's risk behaviors												
Sweetened milk consumption												
No					Reference	–	Reference	–				
Yes					0.99 (0.17, 1.80)	0.017	0.052 (−0.001, 0.106)	0.056				
Sugary beverage consumption												
No					Reference	–	Reference	–				
Yes					0.60 (−0.70, 1.89)	0.367	0.024 (−0.024, 0.073)	0.326				
Child's oral hygiene practice												
Brushing frequency (time/day)												
≤1	Reference	–	Reference	–	Reference	–	Reference	–				
> 1	−0.23 (−1.09, 0.63)	0.599	−0.011 (−0.051, 0.030)	0.606	−0.06 (−0.81, 0.69)	0.875	−0.003 (−0.036, 0.031)	0.875				
Brushing before bed												
Yes	Reference	–	Reference	–	Reference	–	Reference	–				
No	1.15 (0.28, 2.02)	0.01	0.055 (0.002, 0.109)	0.043	1.07 (0.11, 2.03)	0.03	0.049 (−0.001, 0.100)	0.054				
Fluoride toothpaste use												
Yes	Reference	–	Reference	–	Reference	–	Reference	–				
No	−0.63 (−1.69, 0.42)	0.24	−0.026 (−0.069, 0.016)	0.228	−0.23 (−1.43, 0.98)	0.713	−0.010 (−0.060, 0.040)	0.704				

Abbreviation: 95% CI, 95% confidence interval.

## Discussion

All statistical models in Fig. 1 with subsequent results shown in Table 3 consistently identified the positive association between maternal caries during pregnancy and the ECC risk in the offspring by three measures including (1) the positive regression coefficients indicating the positive direction of the effect, (2) the confidence intervals and *p* -value in all models that indicated the observed association was unlikely due to chance, and (3) the AMEs showing the meaningful magnitude of effect. Model 1 estimated the crude effect of maternal caries during pregnancy on the ECC risk. Different sets of covariates were then sequentially adjusted in the multivariable models (Models 2 to 5) following the causal model built upon prior knowledge regarding the relationship of these covariates on the main association between maternal caries and ECC risk.

In Model 2 ( Fig. 1 ), maternal factors comprising maternal age and oral health insurance type were specified as a set of potential confounders as these factors could be common causes of both maternal caries and the ECC risk, as denoted as E←M→D in Fig. 1 . Maternal age could differentiate time at-risk of caries and duration of demineralization/remineralization of tooth 27 that influenced maternal caries occurrence. Maternal age could also influence the ECC risk, especially among teenage mothers who might have limited ability to care for their offspring's oral health. 28 Having oral health insurance could enable access to necessary dental services that influenced both maternal caries status and ECC risk. 29 Nonetheless, despite the influence of maternal age and oral health insurance documented in previous literature, 27 28 29 the estimates adjusted for these factors did not differ considerably from the crude ones in this setting ( Table 3 ).

Although child-related independent factors did not affect maternal dental caries—an event that preceded them, Fig. 1 depicted these factors' positions in various noncausal paths 11 linking maternal caries (E) and the ECC risk (D). Since oral health risk behaviors and oral hygiene care of very young children would primarily rely on their mothers, maternal factors during pregnancy—maternal age and having dental insurance—were therefore assumed to have a causal relationship with the child's risk behaviors and oral hygiene practice in the DAG. Previous evidence supported this assumption. Being a teenage mother—a maternal age characteristic—could be related to disadvantages such as a lack of preparation for motherhood, 30 single parenthood, low educational levels, and socioeconomic deprivation 31 32 that link to a limited capability to provide good care of a child's oral health. 33 34 Lacking oral health insurance could also impede access to prenatal dental care and oral health education necessary to protect a child's oral health. 29 From the assumption regarding the association of the maternal factors during pregnancy (M) with a child's risk behaviors (R) and a child's oral hygiene practice (P), two non-causal paths linking material caries to the ECC risk were conceptualized as E←M→R→D and E←M→P→D. Nonetheless, controlling for maternal factors (M) in Model 2 could block these two non-causal paths. When a child's characteristics (C)—including natal sex and age—were considered for multivariable analysis, they were assumed to have a causal relationship to a child's risk behavior and a child's oral hygiene practice. Previous studies suggested that oral health risk behaviors (e.g., children's preference for sugar) and oral hygiene habits (e.g., tooth brushing frequency) could vary by children's sex 35 36 and age. 37 38 Based on this assumption, the child's sex and age (C) were added to DAG and created two non-causal paths in Fig. 1 as E←M→R←C→D (Model 3) and E←M→P←C→D (Model 4). Model 5 combined the child's risk behaviors and oral hygiene practice and included all extraneous factors between E and D to construct a non-causal path as E←M→R&P←C→D. In Models 3 to 5, the child's risk behaviors (R) and oral hygiene practice (P) were regarded as colliders in epidemiology, and they would block all these non-causal paths and result in no need to control for all extraneous factors between E and D. 11 Nonetheless, multivariable analyses for Models 3 to 5 were undertaken as sensitivity analyses to identify how the effect of maternal caries on the ECC risk changed under various causal assumptions. Collider bias was also avoided in all these models by controlling for M and C simultaneously with the colliders ([R] and [P]).

Results from all univariable and multivariable models consistently confirmed that children born to two categories of mothers with 1–5 and ≥6 carious teeth during pregnancy would develop 4.5–5 and 7.9–8.8 more carious teeth per 100 teeth on average compared with children born to caries-free mothers. Similar findings of the positive association between maternal caries and ECC risk were previously reported. 14 19 39 Untreated maternal caries could be a primary source of cariogenic bacteria acquisition. 17 40 Nonetheless, further process of cariogenesis could be mediated by multiple biological and social factors determining oral health risk behaviors and oral hygiene practice of the child. 14 40

Comparison of ECC risk among children of varying ages and numbers of erupted teeth in a real-world dynamic cohort would be challenging since age would affect caries occurrence. Nonetheless, the measure of ECC risk using π which took into account not only the count number of carious teeth but also the total number of teeth at risk of developing dental caries in this study would allow a rational comparison of risk or probability of caries development across different children. To theoretically exemplify this point, assume that two children both have 4 carious teeth. If only the count number of carious teeth is used as an outcome for comparison, the caries status of these children would be considered identical. Nonetheless, if the first child who is 22 months old with 16 teeth is compared with the second child who is 33 months old with 20 teeth, the first child's π value is (4/16) × 100% or 25%, which is the higher risk of ECC compared with π of (4/20) × 100% or 20% in the second child. The π closer to 100% would show a higher risk of ECC in terms of the proportion of carious teeth from the whole number of erupted teeth.

The findings of this study not only confirmed the positive direction of the association between maternal caries during pregnancy and the ECC risk but also added to the literature the probability of ECC predicted from the status of maternal caries at the first prenatal dental care visit. This suggested the possible role of maternal caries routinely detected during prenatal dental care as a useful clinical risk indicator for ECC. Moreover, the significant effect of maternal caries on the ECC risk in Thai mother–child dyads in this study served as local evidence supporting the national dental public health policy promoting the provision of prenatal dental care as an integral part of antenatal care.

Nonetheless, the generalizability of this study's findings, following the eligibility criteria for matched pair of mother and child in this study, might be limited to mothers and children who both utilized the whole service package from the integrated antenatal care and prenatal oral health care attended by the mothers to the integrated immunization and oral health promotion services attended by the children at this hospital or facilities of simar context. The results might not be generalized to those attending other health facilities with different service contexts such as private clinics and hospitals that were also widely available in Thailand.

## Conclusions

Maternal dental caries during pregnancy was a significant clinical indicator for the increased ECC risk in children. The findings supported the provision of prenatal dental care for mothers to prevent ECC in offspring.
